# The evaluation of L‐arginine solution as a solvent for propolis extraction: The phenolic profile, antioxidant, antibacterial activity, and in vitro bioaccessibility

**DOI:** 10.1002/fsn3.3953

**Published:** 2024-01-11

**Authors:** Gizem Mergen Duymaz, Gamze Duz, Kubra Ozkan, Ayse Karadag, Ozlem Yilmaz, Ayca Karakus, Ozlem Cengiz, Ismail Emir Akyildiz, Gunay Basdogan, Emel Damarlı, Osman Sagdic

**Affiliations:** ^1^ Food Engineering Department Yildiz Technical University Istanbul Turkey; ^2^ Altiparmak Gıda San. ve Tic. A.S. Balparmak R&D Center Istanbul Turkey; ^3^ Department of Chemistry Istanbul Technical University Istanbul Turkey; ^4^ Chemistry Department Marmara University Istanbul Turkey

**Keywords:** bioaccessibility, extraction, flavonoids, LC–MS/MS

## Abstract

Ethanol has been widely used for the extraction of propolis. Due to its certain disadvantages, there has been an ongoing search to find alternative non‐ethanolic extraction solvents. This study aimed to compare the phenolics, antioxidant, and antibacterial activity of propolis extracts prepared with 70% ethanol (EWE), propylene glycol (PGE), and L‐arginine solution (BE). All extracts were subjected to an in vitro simulated digestion procedure, and the phenolic profile of non‐digested and digested samples was determined by using LC–MS/MS. Additionally, the change in total phenolic (TPC), total flavonoid content (TFC), and antioxidant capacities were determined at each digestion phase. TPC and TFC of non‐digested propolis extracts had similar values, although BE showed higher antioxidant capacity (*p* < .05). The amount of TPC reached or transformed at the intestinal stage was higher for BE and PG compared to EWE. BE also provided the highest antioxidant capacity assay in digested samples. The most common phenolics were pinocembrin, pinobanskin, galangin, and CAPE in non‐digested extracts. However, their concentration was drastically reduced by digestion, and their recovery (R%) ranged from 0% to 9.38% of the initial amount detected in the non‐digested extracts. Chrysin was the most bioaccessible flavonoid in all extracts. Among phenolic acids, the highest R% was determined for trans‐cinnamic acid (22.14%) from BE. All extracts showed in vitro inhibitory activity against *Escherichia coli* and *Staphylococcus aureus.* This study suggests that an L‐arginine solution could be used as an alternative solvent to ethanol and propylene glycol for propolis extraction.

## Graphical abstract

This study aimed to compare the phenolics, antioxidant, and antibacterial activity of propolis extracts prepared with propylene glycol (PGE), 70% ethanol (EWE), and L‐arginine solution (BE). All extracts were subjected to an in vitro simulated digestion procedure, and the phenolic profile of non‐digested and digested samples was determined by using LC–MS/MS. Additionally, the change in total phenolic (TPC), total flavonoid content (TFC), and antioxidant capacities were determined at each digestion phase.

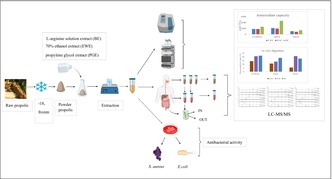

## 1 INTRODUCTION

Propolis is a bee product that possesses a wide range of biological and pharmacological properties, including antioxidant, antimicrobial, antiparasitic activities, anti‐inflammatory effects, immunomodulatory actions, as well as antitumor, anticancer, antiulcer, hepatoprotective, cardioprotective, and neuroprotective properties. (de L. Paula et al., 2021; Dos Santos et al., 2022; Hafshejani et al., 2023; Sagdic et al., 2007).

Its chemical structure is very complicated, with more than 300 different compounds (Ahangari et al., 2018), including plant resins (~50%), waxes (~30%), aromatic and essential oils (~10%), pollens (~5%), and other substances including amino acids, minerals, sugars, phenols, and terpenes (~5%) (Bogdanov, 2017; de L. Paula et al., 2022; Ding et al., 2021). The biological effects of propolis are mainly attributed to phenolic compounds such as phenolic acids, flavonoids, tannins, stilbenes, coumarins, and quinones (Ahangari et al., 2018). Caffeic acid phenyl ester (CAPE), chrysin, galangin, pinobanksin, and pinocembrin were identified as the major active constituents of propolis (Necip et al., 2023; Yen et al., 2017).

Due to its resinous structure, propolis has very low solubility in water; therefore, it needs to undergo an extraction process before it can be digested. The choice of extraction solvent is crucial, as it directly influences the quantity and properties of bioactive compounds in the final product, propolis extract (PE). Maceration with 70%–80% aqueous ethanol has been traditionally used for propolis extraction (Bankova et al., 2021; Santos et al., 2021). However, the presence of alcohol in the extract limits its application in cosmetic, pharmaceutical, nutraceutical, and pediatric products and leads to the exploration of non‐alcoholic extraction solvents. There are different PEs sold on the market as food supplements produced by alternative natural (e.g., water and olive oil) and alcohol derivative extraction solvents (e.g., propylene glycol and glycerin) (Sagdic et al., 2020).

The selectivity of natural extraction solvents may not be proper for the desired phenolic composition in the PE (Kubiliene et al., 2018; Sagdic et al., 2020). Even though glycerin does not have an ADI value, it has been observed that its potential to dissolve propolis is considerably less than ethanol The adverse effects of propylene glycol consumption have been reported previously (Atayoglu et al., 2023; Bakkaloglu & Arici, 2019; Sagdic et al., 2020).

Due to the need for an alcohol‐free extraction approach with natural solvents, Balparmak R&D Center applied an alkaline aqueous extraction medium containing L‐arginine, a semi‐essential amino acid, to prepare PE. L‐arginine is used by the human body for important functions like protein synthesis, the urea cycle, tissue repair, and the immune system (Pahlavani et al., 2017). The external intake of L‐arginine was also associated with a healthy vascular system (Gad, 2010).

Since in vitro digestion assays are easy, inexpensive, and repeatable methods of evaluating the stability of various dietary ingredients against digestive fluids, they have been created as an alternative to in vivo studies. The bioaccessibility of phenolics in the digestive tract is considered a critical factor in interpreting their health benefits. Over the years, there have been a few studies investigating the bioaccessibility of PE phenolics mainly focusing on ethanolic extracts (Ozdal et al., 2019; Yen et al., 2017). The bioaccessibility of phenolics could be influenced by a variety of factors such as the other components in the matrix, the method and the solvent of extraction, and the stage of digestion (Tan et al., 2022). In this study, the phenolic composition, antioxidant capacity, antibacterial activity, and the impact of in vitro gastrointestinal digestion on PE obtained by L‐arginine solution were evaluated and compared to the PE prepared by using ethanol (70%) and mono‐propylene glycol.

## 2 MATERIALS AND METHODS

### 2.1 Chemicals

Mono propylene glycol, 6‐hydroxy‐2,5,7,8‐tetramethyl chroman‐2carboxylic acid (Trolox, 97%), 2,2′‐azinobis (3ethylbenzthiazoline‐6‐sulfonic acid) (ABTS, >98%), 2,4,6‐tris(2‐pyridyl)‐s‐triazine (TPTZ), neocuproine, amylase (A1031), pepsin (P7012), pancreatin (P7545), and bile (B8631) were purchased from Sigma‐Aldrich Ltd (Steinheim, Germany). Methanol Ethyl Acetate NaCl Muller Hinton Agar Muller Hinton Broth (Merck 110293), Nutrient Broth (Merck 105443), Sodium Chloride (Merck 106404), Formic acid (Merck 100264) Copper (II) chloride, ammonium acetate, and L‐Arginine were purchased from Merck KGaA (Darmstadt, Germany).

Phenolic acids and flavonoids used as standards including 3,4‐dimethoxy cinnamic acid, apigenin, chrysin, caffeic acid, caffeic acid phenethyl ester (CAPE), ferulic acid, galangin, gallic acid, genistein, quercetin, kaempferol, isorhamnetin, luteolin, p‐coumaric acid, naringenin, pinobanksin, pinocembrin, and trans‐cinnamic acid were purchased from Sigma‐Aldrich (Steinheim, Germany).

### 2.2 Preparation of propolis extracts (PE)

5 kg of raw propolis were obtained from a local beekeeper in Yalova, Turkey, in February 2020. Impurities were removed by sorting out the propolis sample, which was then frozen at −18°C for 24 h. Frozen propolis was immediately ground into a powder with a mechanical grinder and stored at −18°C until further processing. The propolis extracts (PE) were prepared by using different solvents: 70% ethanol (EWE), mono propylene glycol (PGE), and L‐arginine solution (BE) (pH = 9.0 ± 0.5). The propolis to solvent ratio was kept constant at 1:5 (w:v) for all solvents, and the extraction continued for 24 h in the dark at room temperature. The wax collected at the top was filtered (Whatman no.1), after storing the suspensions at 4°C for 24 h, and the residue was extracted two more times under the same condition. All supernatants were combined, filtered (0.45 μm), filled up to the same volume with their solvents, and stored at 4°C until analysis.

### 2.3 Total phenolic content (TPC) and total flavonoid content (TFC) analysis

The total phenolic content (TPC) of samples was evaluated using Folin–Ciocalteu (FC) method (Singleton et al., 1999). 25 μL of diluted PE were mixed with FC reagent, Na_2_CO_3_ solution (20%), and ultra‐pure water and incubated for 1 h at room temperature, and the absorbance was measured against the blank at 760 nm (Shimadzu 150 UV‐1800 spectrophotometer, Kyoto, Japan). The results were given as mg gallic acid equivalent (GAE)/g raw propolis with a linear range of 0.2–2 mg/mL (*R* ^2^ = .999). The total flavonoid content (TFC) of samples was evaluated by the method of Andrade et al. (2017). The diluted PE was mixed with 0.1 mL of AlCl_3_ and CH_3_COONa solution, the mixture was incubated at room temperature for 30 min, and the absorbance was read against blank at 415 nm. The results were given as mg quercetin (QE)/g raw propolis with a linear range of 0.05–0.5 mg/mL (*R* ^2^ = .999).

### 2.4 Antioxidant capacity assays

To assess the antioxidant capacity, three different assays were conducted with minor modifications (Karadag et al., 2020). The ABTS radical scavenging activity of PEs was given as μmol TE/g raw propolis with a linear range of 40–799 μM (*R* ^2^ = .992). The ferric reducing antioxidant power (FRAP) of samples was given as μmol TE /g raw propolis with a linear range of 10–799 μM (*R* ^2^ = .992). The copper‐reducing antioxidant capacity (CUPRAC) of PE samples was expressed as μmol Trolox Equivalent (TE)/ g raw propolis with a linear range of 100–1998 μM (*R* ^2^ = .993).

### 2.5 Simulated in‐vitro gastrointestinal (GI) digestion

In vitro GI digestion was performed according to Minekus et al. (2014) and Lucas‐González et al. (2018) with minor modifications. The change in antioxidant capacity and the amount of phenolics from the EWE, PGE, and BE were determined by LC/MS–MS at each stage of digestion (oral, gastric, and intestinal). 5 mL of PE were mixed with simulated salivary fluid (pH 7.0) and α‐amylase solution (75 U/mL), CaCl_2_ (0.75 mM), and vortexed for 2 min at 37°C. The oral bolus was mixed with simulated gastric fluid (SGF) CaCl_2_ (0.075 mM), and pepsin solution (2000 U/mL) at a ratio of 1:1 (v:v), pH was adjusted to 3 using HCl. The samples were incubated in a shaking water bath at 37°C for 2 h. Afterward, the gastric chyme was mixed with simulated intestinal fluid, CaCl_2_ (0.3 mM), pancreatin solution (100 U/mL), fresh bile (10 mM) at a ratio of 1:1 (v:v), and pH was adjusted to 7 using NaOH. The segments of dialysis bags (MWCO 12,000 Da) filled with sufficient Na_2_CO_3_ solution (0.1 M) were placed in the SIF medium, and the beakers were kept in a shaking water bath for 2 h at 37°C. The substances that entered the serum (IN) made up the contents of the dialysis bags, while the stuff in the GI tract (OUT) made up the contents outside the bags. The supernatants of the samples taken for the oral, gastric, and intestinal phases were collected after centrifugation at 2480 *g* for 10 min at 4°C. Analysis was also performed on a blank test tube that included all simulated digestive fluids but no samples. All supernatants were lyophilized and kept at −20°C until further analysis. All procedures were done in triplicate.

The effect of in vitro digestion on the phenolics and antioxidant capacity of PE was evaluated by the Recovery percentage (R%) (Lucas‐González et al., 2018) and Bioaccessibility index (BI%) (Minekus et al., 2014) as shown in Equations (1) and (2)

(1) $$R\%=\frac{\text{IN}}{\text{Undigested}}\times 100$$

(2) $$\;\mathrm{BI}\%=\frac{\text{Intestinal phase}\left(\text{IN}+\text{OUT}\right)}{\text{Undigested}}\times 100.$$

### 2.6 LC–MS/MS analysis

The phenolic profiles of undigested and digested samples was determined according to the method of Guzelmeric et al. (2023) by using Waters® Xevo (Waters Corporation, Milford, MA, USA) LC–MS/MS system equipped with a binary pump, a temperature‐controlled column compartment, an autosampler, and a triple‐quadrupole (TQ) MS/MS mass analyzer with ESI ion source. An analytical Cortecs T3 (Waters Corporation Milford, USA) column (2.1 × 150 mm, 1.6 μm) was used for the separation. The mobile phase consisted of two solvents: (A) the acidified water (0.01% acetic acid, v:v), and (B) acetonitrile: methanol (80:20, v:v). The gradient flow rate was 0.25 μL/min. The gradient elution conditions were: 0–1.30 min, 2% B; 1.30–35 min, 2%–55% B; 35–37 min, 55%–95% B; 37–37.01 min, 95%–2% B, and 37.01–40 min, 2% B. The column temperature was 30°C and the injection volume was 5 μL. The eluted samples from LC were analyzed using the ESI‐MS/MS system in multiple reaction monitoring (MRM) mode. The parameters of ESI‐MS/MS were adjusted as follows; the ion source and desolvation temperature were set at 150 and 450°C, respectively. The capillary voltage was 2 kV. Desolvation and cone gas flows were 850 and 50 L/h, respectively. LC–MS/MS data was processed using Waters® Mass‐Lynx software at Target Lynx Program (Waters®). Each phenolic compound was individually injected into the LC–MS/MS system for the determination of retention times, ionization mode, precursor ion energy, capillary voltage, and fragment ions, and optimal MRM transition parameters, such as cone voltage, collision. A stock of standard solutions was prepared in methanol (2 mg/mL), and the calibration curve was prepared at 50, 100, 250, 500, and 1000 μg/L of concentrations. Filtered samples were diluted with acidified methanol: water (1:1, v:v, 0.01% acetic acid) (Table 1).

**TABLE 1:** Phenolic profiles of propolis extracts by LC–MS/MS method.

Compounds	Molecular formula	RT (min.)	Ionization mode	Precursor ion (*m*/*z*)	Product ion(s) (*m*/*z*)	Cone (V)	Collision (eV)	EWE	PGE	BE
								mg/g raw propolis		
3,4 dimethoxy cinnamic acid	C_11_H_12_O_4_	19.1	ESI (−)	206.7	102.7	25	20	4.25 ± 0.52^b^	4.45 ± 0.08^ab^	4.95 ± 0.06^a^
Apigenin	C_15_H_10_O_5_	25.0		269	117.3/149/151	40	30/25/25	1.84 ± 0.06^b^	1.87 ± 0.10^ab^	2.01 ± 0.05^a^
Caffeic acid	C_9_H_8_O_4_	10.9		179	135	25	20	3.88 ± 0.35^ab^	3.88 ± 0.14^ab^	4.37 ± 0.02^a^
CAPE	C_17_H_16_O_4_	32.4		283	179/161/135	25	20	6.41 ± 0.04^b^	6.64 ± 0.11^a^	6.12 ± 0.03^c^
Chrysin	C_15_H_10_O_4_	30.9		253	225/209/151	25	20	4.30 ± 0.10^b^	4.33 ± 0.12^ab^	4.57 ± 0.04^a^
*Trans*‐ferulic acid	C_10_H_10_O_4_	14.7		193	178/149/134	25	20	1.29 ± 0.06^b^	1.22 ± 0.05^b^	1.52 ± 0.11^a^
Galangin	C_15_H_10_O_5_	31.9		269	197/213/227	25	20	7.98 ± 0.13^a^	7.99 ± 0.17^a^	4.30 ± 0.42^b^
Genistein	C1_5_H_10_O_5_	24.7		269	133.2/159.2/224.2 240	40	30/20/25/20	0.33 ± 0.06^a^	0.33 ± 0.02^a^	0.32 ± 0.04^a^
Isorhamnetin	C_16_H_12_O_7_	25.9		315	300	25	20	0.98 ± 0.07^a^	1.05 ± 0.05^a^	0.05 ± 0.02^b^
Kaempferol	C_15_H_10_O_6_	25.4		285	93/151/257	25	20	2.01 ± 0.19^a^	2.13 ± 0.16^a^	0.04 ± 0.01^b^
Luteolin	C_15_H_10_O_6_	22.3		285	133/241/267	25	20	0.48 ± 0.10^a^	0.54 ± 0.16^a^	0.62 ± 0.16^a^
Naringenin	C_15_H_12_O_5_	24.2		271	145/151	25	20	1.07 ± 0.03^b^	1.02 ± 0.02^b^	1.46 ± 0.07^a^
*p*‐coumaric acid	C_9_H_8_O_3_	13.5		163	93/119/147	25	20	1.86 ± 0.07^b^	1.85 ± 0.05^b^	2.22 ± 0.02^a^
Pinobanksin	C_15_H_12_O_5_	24.3		271.2	153/225/253	25	20	6.08 ± 0.14^b^	6.28 ± 0.21^b^	10.65 ± 0.32^a^
Pinocembrin	C_15_H_12_O_4_	31.1		255	151/171/213	25	20	12.54 ± 0.06^ab^	12.78 ± 0.07^a^	11.44 ± 0.20^c^
Quercetin	C_15_H_10_O_7_	22.4		301	150.8/178.9	35	20	1.50 ± 0.03^a^	1.46 ± 0.07^a^	0.06 ± 0.04^b^
*Trans‐*cinnamic acid	C_9_H_8_O_2_	21.1		147	77/102.8	25	20	2.14 ± 0.06^ab^	2.09 ± 0.28^ab^	2.30 ± 0.19^a^
Total								58.94	59.91	57.00

*Note*: Data are expressed as mean ± SD of triplicate measurements. Means with different letters in the same row are significantly different (*p* < .05).

Abbreviations: BE, L‐arginine solution extract of propolis; ESI, electrospray ionization; EWE, 70% ethanolic extract of propolis; PGE, mono propylene glycol extract of propolis; RT, retention time.

### 2.7 Antibacterial activity with disk diffusion method

Strains of *Escherichia coli* O157 ATCC 700728 and *Staphylococcus aureus* ATCC 25923 were obtained from the American Type Culture Collection. The in vitro inhibitory activities of PE were determined by the disc diffusion method (Sagdic et al., 2007). They were inoculated with nutrient agar for 24 h, at 37°C. They were adjusted against 0.5 Mc Farland turbidity standard tubes (1 × 10^8^). 6 mm sterilized discs (Bioanalyse Antimicrobial susceptibility test discs) were impregnated with 30 μL of extracts (without dilution) and placed on Muller Hilton agar plates, and incubated for 24 h, at 37°C. Inhibition zones (mm) on the medium were evaluated. All tests were performed in triplicate. The antibacterial activity of each solvent used for extraction was also assessed for control. The chloramphenicol disc (HIMEDIA Chloramphenicol C300 SD006‐SCT) was used as a positive control.

### 2.8 Statistical analysis

The data were expressed as the mean ± standard deviation (SD), analyzed by one‐way analysis of variance (ANOVA), and Tukey's post hoc test was used for comparison of the means using SPSS Statistics Software (IBM version 20, USA). All experiments were carried out in triplicate.

## 3 RESULTS AND DISCUSSION

### 3.1 Total phenolic and total flavonoid content

The total phenolic content (TPC) and the total flavonoid content (TFC) of PEs are given in Table 2. TPC of PEs ranged between 109.87 ± 2.64 and 116.36 ± 3.08 mg GAE/g raw propolis (*p* > .05). The TFC of PEs was not significantly different and ranged between 45.67 ± 0.06 and 47.54 ± 2.05 mg QE/g raw propolis. Kubiliene et al. (2015) also reported no significant differences in the TPC between PE obtained by PGE and ethanol (70%). Our results were in the range of the study of Ozdal et al. (2019), who compared TPC (27.48–199.70 mg GAE/g) and TFC (30.73–291.75 mg QE/g) of ethanolic extracts of 11 propolis samples collected from different regions. The content of propolis may vary depending on the plant species that honeybees collected the resin. The TPC and TFC values determined in our study were also in the range of Xue et al. (2016), who stated that the TPC and TFC values of the ethanolic extracts of propolis samples from Brazil, Australia, China, and South Korea ranged from 48.5 ± 4.08 to 238.9 ± 4.61 mg GAE/g and 23.5 ± 0.15 to 53.0 ± 0.22 mg QE/g, respectively. Ding et al. (2021) reported that TPC and TFC values of Chinese propolis collected from five regions was ranged from 72.02 to 155.95 mg GAE/g, and 129.68 to 412.83 mg QE/g of ethanolic extracts, respectively. Whereas Iranian propolis samples showed TPC and TFC values ranged from 26.59 to 221.38 mg GAE/g and from 4.8 to 100.03 mg QE per g of ethanolic extract, respectively (Hafshejani et al., 2023).

**TABLE 2:** Total phenolic, flavonoid contents, and antioxidant capacities of propolis extracts.

	EWE	PGE	BE
TPC	112.08 ± 2.24^a^	109.87 ± 2.64^a^	116.36 ± 3.08^a^
TFC	47.54 ± 2.05^a^	46.41 ± 1.46^a^	45.67 ± 0.067^a^
CUPRAC	1837.81 ± 46.67^c^	1803.44 ± 92.07^c^	2592.29 ± 43.90^a^
ABTS	2150.92 ± 132.55^bc^	1940.20 ± 126.72^c^	4593.52 ± 97.55^a^
FRAP	1089.65 ± 57.14^b^	861.88 ± 38.19^c^	1397.29 ± 53.16^a^

*Note*: Data are expressed as mean ± SD of triplicate measurements. Means with different letters in the same row are significantly different (*p* < .05).

Abbreviations: ABTS, 2,2′‐azino‐bis (3‐ethylbenzothiazoline6‐sulphonic acid); BE, L‐arginine solution extract of propolis; CUPRAC, Copper reducing antioxidant capacity; EWE, 70% ethanolic extract of propolis; PGE, mono propylene glycol extract of propolis; FRAP, ferric reducing antioxidant power expressed as μmol TE/g raw propolis; TFC, total flavonoid contents expressed as mg QE /g raw propolis; TPC, total phenolic contents expressed as mg GAE/g raw propolis.

The aqueous extract of propolis yielded substantially lower TPC and TFC than those of the ethanolic extract in the study of Kubiliene et al. (2018) and Mokhtar (2019). In our study, extraction with L‐arginine solution (BE) yielded similar TPC and TFC values compared to EWE and PGE. This situation could be attributed to the change in ionic strength of the water, which can affect the solubility of compounds (Chong & Chua, 2020).

### 3.2 Total antioxidant capacity

It was stated that a single assay would not be enough to assess the results for complex matrices that include more than one compound possessing multiple antioxidant mechanisms (Karadag et al., 2009). Therefore, in this study, the results of three different assays (CUPRAC, ABTS, and FRAP) were evaluated (Table 2). Each method serves a different mechanism of the reaction, for example, CUPRAC and FRAP were classified as electron transfer (ET) based methods whereas ABTS assay was classified as a hydrogen atom transfer (HAT) based method (Karadag et al., 2009).

The CUPRAC, ABTS and FRAP values of our samples varied from 1803.44 to 2592.29, 1940.20 to 4593.52, and 861.88 to 1397.29 μmol TE/g raw propolis, respectively. Our ABTS result for PGE and EWE was in the similar range of the 19 PE analyzed by Bonvehí and Gutiérrez (2011) whereas the ABTS, CUPRAC and FRAP values of our ethanolic extract of propolis (EWE) were higher than those determined by Ozdal et al. (2019). Although their TPC and TFC values were similar, BE showed significantly higher antioxidant capacity values than other propolis extracts. The individual phenolic compounds in the extracts depending on the solvent might also display different antioxidant capacities. For example, compared with the other propolis extracts, BE had a significantly higher amount of ferulic acid, p‐coumaric acid, naringenin, and pinobaksin (Table 1). All antioxidant capacity assays were conducted in an aqueous medium, therefore the solubility of the extracts in the reaction medium should be also another important parameter to consider. When the propolis extracts were mixed with the reaction medium of antioxidant assays, the BE sample would have the advantage of enhanced solubility in the reaction medium due to the aqueous nature of its solvent. The pH of BE was around 9, it was reported that the antioxidant capacity of propolis extracts was increased when the extraction was applied in an alkaline condition (Yeo et al., 2015).

### 3.3 Analysis of individual phenolics in propolis extracts by LC–MS/MS

The individual phenolics and their concentration in the propolis extracts (EWE, PGE, and BE) were given in Table 1. The phenolic profiles of samples were typical for poplar propolis with the presence of flavonoids such as CAPE, pinocembrin, pinobaksin, galangin, apigenin, chrysin, quercetin, and phenolic acids such as ferulic acid, p‐coumaric acid, and cinnamic acid (Guzelmeric et al., 2023; Ozdal et al., 2019).

The sum of the amounts of identified phenolic acids and flavonoids in the three extracts was between 57.00 and 59.91 mg/g of raw propolis, and pinocembrin and pinobanksin were the most abundant phenolics, ranging from 11.44 to 12.78 mg/g and 6.08 to 10.65 mg/g, respectively. Pinobanksin and pinocembrin were reported to be the main flavanones of the European propolis (Hegazi et al., 2000). While pinobanksin content was higher in BE, pinocembrin was determined to be higher in EWE and PGE. The elevated concentrations of galangin and CAPE were also detected in all extracts varying from 4.30 to 7.99 and 6.12 to 6.64 mg/g, respectively. Ozdal et al. (2019) determined the concentration of pinobanksin and CAPE ranged from 0.24 to 3.84 mg/g and 0.4 to 2.86 mg/g, respectively, in propolis ethanol extracts collected from different parts of Turkey. While phenolic acids (*p*‐coumaric acids, caffeic, and ferulic), which are more soluble in water, were identified at higher concentrations in BE, the level of galangin, isorhamnetin, kaempferol, and quercetin was lower compared with EWE and PGE samples. This could be due to the instability of the flavonol group at high pH compared to other subgroups of flavonoids such as flavanone, flavanol, and flavone. There are three main structural points that determine the reactivity of flavonoids: (i) the C3‐OH group in the C‐ring, (ii) the catechol moiety in the B‐ring, and (iii) the C2 = C3 bond in the C‐ring (Jurasekova et al., 2014). Galangin, isorhamnetin, kaempferol, and quercetin have both C3‐OH and C2 = C3 double bonds in their structure. In addition to these two groups, quercetin also has a catechol group, which makes it very sensitive to higher pH.

### 3.4 In vitro bioaccessibility of TPC, TFC, and individual phenolics of propolis extracts

During digestion through the simulated oral, gastric fluid, and intestinal fluid phases, the TPC, TFC, and antioxidant capacity of the samples were determined at the end of each digestion stage (Table 3). In all samples, the amount of TPC and TFC released in the oral phase was reduced in the following gastric stage, most probably owing to the low solubility of phenolics in the gastric fluid, at which a low pH value causes the protonation of phenolic compounds and alters them to a more hydrophobic nature. Yen et al. (2017) revealed that the maximum amount of total phenolics and flavonoids in propolis extracts of ethanol, water, and glycerol were noted in the oral stage of digestion. The high enzymatic activity and dialysis filtration in the following digestion phases could lower the phenolic and flavonoid contents (Yen et al., 2017). Consequently, the antioxidant capacity values of all propolis extracts were reduced in the gastric stage, and this reduction was less prominent in BE.

**TABLE 3:** Total phenolic, flavonoid contents, and antioxidant capacities of propolis extracts during in vitro gastrointestinal digestion.

Analyses	Oral	Gastric	Intestinal		R%	BI%
			OUT	IN		
TPC						
EWE	62.22 ± 2.57^a^	23.87 ± 2.50^c^	39.14 ± 2.68^b^	3.46 ± 1.05^d^	3.08 ± 0.91^AB^	37.48 ± 1.14^B^
PGE	76.72 ± 3.57^a^	22.14 ± 7.11^c^	49.58 ± 3.27^b^	2.53 ± 0.65^d^	2.28 ± 0.44^B^	46.85 ± 1.11^A^
BE	55.35 ± 2.26^a^	35.86 ± 1.37^b^	47.90 ± 8.51^ab^	5.18 ± 0.01^c^	4.52 ± 0.04^A^	45.01 ± 4.37^A^
TFC						
EWE	32.09 ± 2.96^a^	14.99 ± 6.60^b^	16.73 ± 1.46^b^	2.21 ± 0.48^c^	5.65 ± 1.14^A^	73.74 ± 4.58^A^
PGE	30.34 ± 2.04^a^	11.18 ± 6.16^b^	11.83 ± 1.24^b^	1.85 ± 0.52^c^	4.63 ± 0.88^A^	53.88 ± 0.27^B^
BE	11.62 ± 1.04^a^	9.66 ± 1.56^b^	12.51 ± 0.97^a^	1.98 ± 0.27^c^	5.20 ± 0.41^A^	58.15 ± 1.92^B^
CUPRAC						
EWE	672.76 ± 22.16^a^	251.61 ± 4.61^b^	231.38 ± 12.90^b^	21.07 ± 0.37^c^	1.15 ± 0.03^B^	14.19 ± 0.82^B^
PGE	377.21 ± 6.13^a^	314.75 ± 3.28^b^	375.75 ± 11.25^a^	18.09 ± 1.04^c^	1.01 ± 0.11^B^	20.85 ± 0.19^A^
BE	520.11 ± 31.12^a^	339.00 ± 6.21^b^	506.26 ± 10.34^a^	38.07 ± 1.66^c^	1.47 ± 0.08^A^	21.56 ± 0.15^A^
ABTS						
EWE	423.70 ± 25.05^a^	168.26 ± 33.60^b^	206.18 ± 26.54^b^	17.83 ± 1.81^cC^	0.83 ± 0.11^C^	10.92 ± 1.21^C^
PGE	544.57 ± 62.84^a^	93.72 ± 5.41^c^	200.99 ± 15.10^b^	53.17 ± 3.06^cB^	2.75 ± 0.31^A^	13.63 ± 0.91^B^
BE	601.74 ± 36.96^b^	396.80 ± 6.83^c^	750.75 ± 67.08^a^	60.94 ± 0.42^dA^	1.35 ± 0.01^B^	18.64 ± 0.52^A^
FRAP						
EWE	318.18 ± 13.96^a^	133.50 ± 3.36^b^	55.72 ± 2.48^c^	5.01 ± 0.33^d^	0.46 ± 0.04^B^	5.59 ± 0.44^C^
PGE	198.84 ± 8.02^a^	64.95 ± 5.35^c^	183.47 ± 5.07^b^	7.68 ± 0.58^d^	0.92 ± 0.08^A^	21.07 ± 0.33^A^
BE	216.45 ± 12.81^a^	162.91 ± 2.98^b^	216.45 ± 19.49^a^	10.80 ± 0.80^c^	0.77 ± 0.08^A^	17.67 ± 0.37^B^

*Note*: Data are expressed as mean ± SD of triplicate measurements. Different lowercase letters in the same row for each propolis extract among digestion phases are significantly different (*p <* .05). Different capital letters on the same column are significantly different (*p <* .05) among propolis extracts. R% = (IN/Indigested) * 100, BI% = ((IN + OUT)/Indigested) * 100.

Abbreviations: ABTS, 2,2′‐azino‐bis (3‐ethylbenzothiazoline6‐sulphonic acid); BE, L‐arginine solution extract of propolis; CUPRAC, copper reducing antioxidant capacity; EWE, 70% ethanolic extract of propolis; FRAP, ferric reducing antioxidant power expressed as μmol TE/g raw propolis; PGE, mono propylene glycol extract of propolis; TFC, total flavonoid contents expressed as mg QE/g raw propolis; TPC, total phenolic contents expressed as mg GAE/g raw propolis.

At the intestinal phase (IN+OUT), compared to the previous stage of digestion, the increase in TPC and TFC could be related to the presence of digestive enzyme and bile salt that could also affect the wax residues in the extracts, therefore increasing the release of some phenolics partitioned in the wax residue. Additionally, the higher level of pH at this stage could provide higher solubility for propolis compounds. Except for the FRAP value of EWE, all samples had higher antioxidant capacity values in the intestinal stage (Table 3). The measured value in the IN fraction was lower than the OUT fraction, which can be due to the dialysis filtration process (Yen et al., 2017). In our study, the TPC losses in post‐gastric digestion (PG) were 78.70%, 79.84%, and 69.18% for EWE, PGE, and BE, respectively, whereas in the study of Turkut et al. (2019), TPC losses in post‐gastric digestion were 85.72% and 92.11% for ethanol and mono‐propylene glycol propolis extracts, respectively. Compared with EWE, BE and PGE provided a higher amount of TPC at the intestinal phase, and 37.48%–46.85% of the initial TPC of the propolis extracts were accounted for in the intestinal phase (BI%). Only 2.28%–4.52% of the TPC determined in the non‐digested extracts could pass through the dialysis membrane and be considered to enter serum (R%) after simulated GI digestion (Table 3). The BI% of TPC for propolis extracts prepared by water, ethanol, and propylene glycol was between 17.42% and 29.59% (Turkut et al., 2019). In another study, the BI% of TPC ranged from 3.6% to 33.05% for ethanol extracts of 11 different Turkish propolis samples (Ozdal et al., 2019). EWE provided the highest TFC at the intestinal phase. The amount of TFC recovered in the intestinal phase (BI%) ranged from 53.88% to 73.74% for all extracts and compared with the initial amount in the non‐digested extracts 4.63% to 5.20% of total flavonoids were recovered (R%) in the IN phase of digestion, that was not significantly different among extracts. The BI% of TFC in ethanolic extracts of 11 different propolis was reported to change from 1.42% to 136% by Ozdal et al. (2019).

While considering the antioxidant capacities of the compounds reached or transformed in the intestinal stage (IN+OUT) and those entered in the serum (IN), the BE sample provided the highest values by each assay. When calculating their proportion to the initial value in the non‐digested sample extracts (BI%), the results differed depending on the assay. In all extracts, the compounds at the intestinal stage provided only 5.59%–21.6% (BI%) of the initial antioxidant capacities of the related extracts. While with the CUPRAC and ABTS assays, the highest BI% values were determined in BE, it was highest in PGE with the FRAP assay. While considering the antioxidant capacity of the compounds that could pass through the dialysis membrane, the lowest recovery (R%) was detected in EWE with FRAP assay, and the highest recovery was calculated in BE with CUPRAC assay. The change of individual phenolics at each digestion stage determined by LC–MS/MS was given in Table 4. In the non‐digested extracts, pinocembrin, pinobanskin, galangin, and CAPE (Table 1) were detected at higher levels in EWE and PGE, but not in BE. However, in all extracts, their concentration was drastically reduced at the end of the intestinal digestion stage, the recovery (R%) of those flavonoids ranged from 0% to 9.38% of the initial amount determined in the non‐digested samples, and the highest bioaccessibility (BI%) of those compounds was recorded for the EWE. Regarding to the other flavonoids, at the end of digestion, chrysin became the highest bioaccessible flavonoid in all extracts, while quercetin, isorhamnetin, and kaempferol were detected in neither of the samples. Apigenin and genistein were both detected at similar concentrations in all extracts. Luteolin was not detected in PGE, and naringenin was not detected in BE at the end of intestinal digestion. Among all phenolics, the highest recovery (R%) was determined for some phenolic acids of the BE sample, namely 3,4 dimethoxy cinnamic acid (21.16%), ferulic acid (10.34%), *p*‐coumaric acid (13.30%), and trans‐cinnamic acid (22.14%). The difference in the bioaccessibility of phenolics of propolis extracts could be affiliated with their structures. For example, Li et al. (2023) stated that the average bioaccessibility of phenolic acids was higher than that of flavonoids and the bioaccessibility rate decreased with the increase in the number of hydroxyl substitutions among hydroxybenzoic phenolic acids. In our sample, the bioaccessibility of caffeic acid was also lower than that of *p*‐coumaric acid. The increase in the concentrations of phenolic acids induced by in vitro digestion conditions might be associated with the cleavage of higher molecular structures to free phenolic acids, for example, the main hydroxycinnamic acids found in the diet, caffeic, *p*‐coumaric, trans‐cinnamic, and ferulic acids were naturally found as glycosides or esters of quinic acid (Stalmach, 2014). Besides the simple phenolic acids, the presence of some esterified and/or methylated derivatives, such as *p*‐coumaric acid methyl ester, *p*‐coumaric acid isoprenyl ester, caffeic acid esters such as caffeic acid phenylethyl ester (CAPE), caffeic acid isoprenyl and benzyl esters, 3,4‐dimethyl‐caffeic acid (DMCA) were reported in propolis extracts (Falcão et al., 2013; Ozdal et al., 2019). According to Cianciosi et al. (2020), phenolic acids are thought to be more stable in the digestive process than flavonoids. Compared with the higher in vitro bioaccessibility values for honey phenolic acids (*p*‐coumaric, ferulic, and syringic acids, 15.2%, 20.0%, and 25.3%, respectively), very low bioaccessibility values for pinocembrin (0.5%), naringenin (0%), and quercetin (0%) were reported (Seraglio et al., 2021).

**TABLE 4:** Change in the individual phenolics of propolis extracts during in vitro gastrointestinal digestion.

		Oral	Gastric	Intestinal		R%	BI%
				IN	OUT		
*Phenolics (mg/g raw propolis)*							
3,4 dimethoxy cinnamic acid	EWE	9.54 ± 0.77^a^	6.77 ± 1.27^a^	0.53 ± 0.09^b^	7.71 ± 0.54^a^	12.41 ± 1.02^B^	165.29 ± 2.82^B^
	PGE	8.47 ± 0.03^a^	3.89 ± 0.93^b^	0.29 ± 0.10^c^	6.51 ± 0.38^a^	8.18 ± 0.20^C^	153.80 ± 3.03^B^
	BE	9.24 ± 0.27^a^	5.99 ± 0.42^b^	1.04 ± 0.04^c^	8.93 ± 0.08^a^	21.16 ± 0.56^A^	200.29 ± 3.96^A^
Apigenin	EWE	1.89 ± 0.09^a^	0.30 ± 0.03^c^	0.05 ± 0.01^d^	0.95 ± 0.05^b^	3.59 ± 0.06^A^	54.82 ± 3.20^A^
	PGE	1.62 ± 0.10^c^	0.35 ± 0.12^a^	0.04 ± 0.01^a^	0.95 ± 0.08^b^	2.06 ± 0.43^A^	56.27 ± 3.97^A^
	BE	1.12 ± 0.01^c^	0.11 ± 0.01^ab^	0.05 ± 0.02^a^	0.42 ± 0.15^b^	2.71 ± 0.76^A^	28.67 ± 1.24^B^
Caffeic acid	EWE	3.64 ± 0.29^a^	4.42 ± 1.68^a^	nd	0.04 ± 0.01^b^	nd	nd
	PGE	3.18 ± 0.07^a^	1.90 ± 0.57^b^	0.01 ± 0.00^c^	0.25 ± 0.03^c^	0.34 ± 0.03^A^	6.93 ± 0.80^A^
	BE	nd	3.88 ± 0.50^a^	nd	3.60 ± 0.81^a^	nd	nd
CAPE	EWE	3.80 ± 0.28^a^	0.83 ± 0.14^b^	0.02 ± 0.01^c^	0.85 ± 0.36^b^	0.51 ± 0.00^B^	17.41 ± 0.16^B^
	PGE	4.75 ± 0.17^a^	1.10 ± 0.34^b^	0.05 ± 0.04^c^	1.24 ± 0.09^b^	1.12 ± 0.03^A^	19.19 ± 0.51^A^
	BE	0.27 ± 0.01^ab^	0.05 ± 0.01^b^	nd	nd	nd	nd
Chrysin	EWE	4.23 ± 0.26^a^	0.40 ± 0.12^c^	0.34 ± 0.11^c^	3.00 ± 0.04^b^	10.77 ± 0.17^A^	78.75 ± 1.52^A^
	PGE	3.67 ± 0.14^a^	1.01 ± 0.30^b^	0.27 ± 0.04^c^	3.26 ± 0.15^a^	6.25 ± 0.74^B^	82.85 ± 1.79^A^
	BE	3.76 ± 0.10^a^	0.53 ± 0.05^c^	0.31 ± 0.07^c^	1.89 ± 0.51^b^	6.77 ± 1.42^B^	56.10 ± 0.84^B^
*Trans*‐ferulic acid	EWE	1.41 ± 0.18^a^	1.42 ± 0.47^a^	0.04 ± 0.01^B^	0.88 ± 0.09^a^	2.85 ± 0.87^B^	69.62 ± 4.85^B^
	PGE	1.25 ± 0.02^a^	0.69 ± 0.20^b^	0.04 ± 0.01^c^	0.89 ± 0.12^ab^	2.80 ± 0.93^B^	84.34 ± 1.78^B^
	BE	nd	1.62 ± 0.18^a^	0.16 ± 0.01^b^	1.77 ± 0.01^a^	10.31 ± 0.97^A^	124.34 ± 10.14^A^
Galangin	EWE	7.01 ± 0.51^a^	0.72 ± 0.09^b^	0.02 ± 0.00^b^	0.04 ± 0.01^b^	0.30 ± 0.01^C^	0.78 ± 0.02^C^
	PGE	7.97 ± 0.33^a^	1.65 ± 0.59^b^	0.04 ± 0.02^c^	0.22 ± 0.01^c^	0.67 ± 0.01^A^	3.33 ± 0.15^A^
	BE	1.07 ± 0.01^a^	0.07 ± 0.01^bc^	0.02 ± 0.00^c^	0.10 ± 0.03^b^	0.49 ± 0.06^B^	2.65 ± 0.82^AB^
Genistein	EWE	0.40 ± 0.02^a^	0.06 ± 0.01^c^	0.01 ± 0.00^d^	0.20 ± 0.02^b^	3.77 ± 0.46^A^	70.00 ± 4.56^A^
	PGE	0.32 ± 0.02^a^	0.08 ± 0.03^c^	0.01 ± 0.00^d^	0.20 ± 0.01^b^	2.76 ± 0.67^A^	66.88 ± 4.04^A^
	BE	0.23 ± 0.01^a^	0.02 ± 0.00^b^	0.01 ± 0.00^b^	0.09 ± 0.04^b^	4.22 ± 0.96^A^	44.53 ± 1.44^B^
Isorhamnetin	EWE	0.21 ± 0.01^a^	0.15 ± 0.03^a^	nd	nd		
	PGE	0.93 ± 0.05^a^	0.18 ± 0.07^b^	nd	nd	nd	nd
	BE	0.01 ± 0.00^a^	nd	nd	nd		
Kaempferol	EWE	0.57 ± 0.17^a^	0.34 ± 0.02^a^	nd	nd	nd	nd
	PGE	1.39 ± 0.14^a^	0.32 ± 0.15^b^	nd	nd	nd	nd
	BE	nd	nd	nd	nd		nd
Luteolin	EWE	0.38 ± 0.05^a^	0.16 ± 0.07^bc^	0.01 ± 0.00^c^	0.18 ± 0.04^b^	2.29 ± 0.50^A^	46.44 ± 0.93^A^
	PGE	0.34 ± 0.06^a^	0.11 ± 0.02^c^	nd	0.17 ± 0.02^b^	nd	nd
	BE	0.19 ± 0.03^a^	0.04 ± 0.02^bc^	0.01 ± 0.00^c^	0.08 ± 0.01^b^	1.63 ± 0.04^A^	13.06 ± 0.98^B^
Naringenin	EWE	1.63 ± 0.03^a^	0.79 ± 0.13^b^	0.07 ± 0.01^c^	0.77 ± 0.07^b^	6.63 ± 1.68^A^	76.95 ± 2.71^A^
	PGE	1.04 ± 0.04^a^	0.51 ± 0.12^b^	0.07 ± 0.01^c^	0.16 ± 0.01^c^	6.83 ± 1.21^A^	23.13 ± 0.02^B^
	BE	nd	nd	nd	nd	nd	nd
*p*‐coumaric acid	EWE	2.23 ± 0.11^a^	2.21 ± 0.66^a^	0.10 ± 0.03^b^	1.89 ± 0.16^a^	5.43 ± 1.69^B^	98.69 ± 1.90^B^
	PGE	1.77 ± 0.07^a^	1.34 ± 0.28^a^	0.05 ± 0.02^b^	1.52 ± 0.20^a^	2.73 ± 0.83^B^	93.40 ± 2.41^B^
	BE	2.46 ± 0.25^ab^	2.33 ± 0.32^b^	0.29 ± 0.02^c^	3.22 ± 0.24^a^	13.30 ± 1.06^A^	157.71 ± 7.79^A^
Pinobanksin	EWE	9.69 ± 0.04^a^	4.66 ± 0.76^b^	0.49 ± 0.09^c^	4.38 ± 0.57^b^	9.38 ± 0.26^A^	77.94 ± 7.06^A^
	PGE	5.85 ± 0.26^a^	2.82 ± 0.69^b^	0.52 ± 0.10^c^	1.00 ± 0.04^c^	8.35 ± 1.31^A^	24.47 ± 1.98^B^
	BE	nd	nd	nd	nd	nd	nd
Pinocembrin	EWE	13.15 ± 0.46^a^	2.35 ± 0.09^c^	0.72 ± 0.12^d^	7.05 ± 0.29^b^	5.72 ± 1.36^A^	61.94 ± 3.67^A^
	PGE	12.31 ± 0.32^a^	3.52 ± 1.09^b^	0.54 ± 0.13^c^	2.92 ± 0.07^bc^	4.24 ± 1.00^A^	27.19 ± 0.26^B^
	BE	0.12 ± 0.01^a^	0.07 ± 0.01^a^	nd	0.07 ± 0.02^a^	nd	nd
Quercetin	EWE	nd	0.40 ± 0.14^a^	nd	nd		
	PGE	0.93 ± 0.03^a^	0.34 ± 0.12^b^	nd	nd	nd	nd
	BE	nd	nd	nd	nd		
*Trans*‐cinnamic acid	EWE	5.06 ± 0.21^a^	3.06 ± 0.89^bc^	0.26 ± 0.03^c^	4.01 ± 0.05^b^	12.30 ± 1.54^B^	193.79 ± 1.97^B^
	PGE	4.01 ± 0.24^a^	2.18 ± 0.54^b^	0.17 ± 0.04^c^	3.21 ± 0.07^a^	7.75 ± 0.46^B^	138.04 ± 1.56^C^
	BE	4.37 ± 0.35^a^	2.95 ± 0.15^b^	0.52 ± 0.01^c^	4.41 ± 0.03^a^	22.14 ± 2.77^A^	225.54 ± 0.40^A^

*Note*: Data are expressed as mean ± SD of triplicate measurements. Different lowercase letters in the same row for each propolis extract among digestion phases are significantly different (*p <* .05). Different capital letters on the same column are significantly different (*p <* .05) among propolis extract. R% = (IN/Indigested) * 100, BI% = ((IN + OUT)/Indigested) * 100.

Abbreviations: BE, L‐arginine solution extract of propolis; EWE, 70% ethanolic extract of propolis; nd: not determined; PGE, mono propylene glycol extract of propolis.

### 3.5 Antibacterial activity

The antibacterial activity of propolis has been associated to some of its constituents such as the presence of some flavonoids such as galangin, pinocembrin, and pinobanksin at high concentrations (Ding et al., 2021). All propolis extracts showed antibacterial effects against Gram‐negative and Gram‐positive bacterial pathogens (Table 5). Compared with the positive control, chloramphenicol, all propolis extracts showed weaker antimicrobial activity against both *E. coli* and *S. aureus*.

**TABLE 5:** Antibacterial activity of propolis extracts.

Samples	Average inhibition zones (mm)	
	*Escherichia coli*	*Staphylococcus aureus*
BE	9.0 ± 0.09	13.9 ± 0.63
PGE	8.0 ± 0.24	11.1 ± 0.16
EWE	11.4 ± 0.71	19.4 ± 3.13
Chloramphenicol	32.3 ± 0.05	30.6 ± 1.50
BE blank	0.0 ± 0.00	0.0 ± 0.00
PGE blank	0.0 ± 0.00	0.0 ± 0.00
EWE blank	8.8 ± 0.24	11.2 ± 0.38

*Note*: The results are mean ± standard deviation of replicates (*n* = 3). Chloramphenicol was used as the positive control.

Abbreviations: EWE, 70% ethanolic extract of propolis; PGE, mono propylene glycol extract of propolis; BE, L‐arginine solution extract of propolis.

The inhibition zones provided by our samples ranged from 11.1 to 19.4 mm against *S. aureus* and 8.0 to 11.4 against *E. coli*. Compared with *E. coli*, *S. aureus* was reported to be more sensitive to propolis extracts (Bayram et al., 2017). The inhibition zone of ethanolic propolis extracts obtained in Serbia was in the range of 9–13 mm against *S. aureus*, while only two ethanolic propolis extracts out of 13 samples showed an antibacterial effect against *E. coli* (Stepanović et al., 2003). Muli and Maingi (2007) reported that the propolis extracts from three regions of Kenya showed the inhibition zone ranged from 8 to 9.5 mm to 9 to 10 mm against *S. aureus* and *E. coli*, respectively.

Although the antimicrobial effect of the EWE sample seems to be higher, it should be kept in mind that the solvent of this extract (70% of ethanol), provided the antimicrobial effect by itself (Table 5), whereas the other solvents used in PGE and BE do not have antibacterial effects on their own, and the entire effect was due to propolis extract.

## 4 CONCLUSION

Although the broad spectrum of pharmacological and biological properties of propolis has been studied and well‐documented in many different studies, during its oral digestion, the choice of extraction solvent becomes a limiting and important factor due to the solubility of the desired bioactive compounds and its safe and extensive use in different consumer products. This study showed that as an alternative to ethanol and propylene glycol, which are the most commonly used solvents on the market, the aqueous L‐arginine solution can be used as an extraction solvent in terms of phenolic composition, antioxidant, and antimicrobial activity. However, when subjected to in vitro simulated digestion conditions, there was a very drastic reduction of all individual phenolics regardless of the extraction solvent used. Therefore, an efficient delivery system should be proposed for propolis extracts that would also protect the phenolics through digestion.

## AUTHOR CONTRIBUTIONS

**Gizem Mergen Duymaz:** Data curation (equal); formal analysis (equal); methodology (equal); validation (equal); writing – original draft (equal); writing – review and editing (equal). **Gamze Duz:** Data curation (equal); formal analysis (equal); validation (equal); writing – original draft (equal); writing – review and editing (equal). **Kubra Ozkan:** Data curation (equal); formal analysis (equal); writing – original draft (equal); writing – review and editing (equal). **Ayse Karadag:** Conceptualization (equal); methodology (equal); validation (equal); writing – original draft (equal); writing – review and editing (equal). **Ozlem Yilmaz:** Data curation (equal); resources (equal). **Ayca Karakus:** Data curation (equal); resources (equal). **Ozlem Cengiz:** Data curation (equal); resources (equal). **Ismail Emir Akyildiz:** Data curation (equal); resources (equal). **Gunay Basdogan:** Funding acquisition (equal); writing – review and editing (equal). **Emel Damarlı:** Funding acquisition (equal). **Osman Sagdic:** Conceptualization (equal); methodology (equal).

## FUNDING INFORMATION

This study did not receive any grant from funding agencies in the public, commercial, etc.

## CONFLICT OF INTEREST STATEMENT

There are no conflicts of interest.

## Data Availability

The authors confirm that the data supporting the findings of this study are available within the article. The datasets generated during and/or analyzed during the current study are available from the corresponding author on reasonable request.
